# Kidney-Derived ECM Hydrogels as Cell Delivery Devices

**DOI:** 10.1021/acsami.4c15873

**Published:** 2025-03-10

**Authors:** Ana M. Rodrigues, Sara Gimondi, Rita Quinteira, Helena Ferreira, Albino Martins, Nuno M. Neves

**Affiliations:** † 226382 3B’s Research Group, I3Bs - Research Institute on Biomaterials, Biodegradables and Biomimetics of University of Minho, AvePark, Parque de Ciência e Tecnologia, Rua Ave 1, Edificio 1 (Sede), Barco, Guimarães 4805-694, Portugal; ‡ ICVS/3B’s - PT Government Associate Laboratory, Guimarães 4710-057, Portugal

**Keywords:** decellularized kidney, extracellular matrix, collagen, hydrogel, adipose-derived mesenchymal stem cells, renal differentiation

## Abstract

Chronic kidney disease (CKD) represents a significant global health challenge, as emphasized by its increasing prevalence and limited treatment options. Stem cell-based therapies are promising alternatives for CKD treatment. In particular, adipose-derived mesenchymal stem cells (ASCs) have emerged as an attractive candidate cell source. However, challenges in optimizing stem cell delivery and survival upon implantation persist. The inclusion of stem cells in hydrogels addresses these challenges by providing mechanical support coupled to bioactive cues essential for kidney regeneration. In particular, hydrogels derived from a decellularized kidney extracellular matrix (dKECM) offer a biomimetic platform rich in native and important renal components. Herein, we investigate the performance of dKECM hydrogels with respect to the differentiation of ASCs toward kidney-specific phenotypes. First, dKECM hydrogels were characterized and compared with commercially available collagen I hydrogels, which are typically used for this therapeutic application. Subsequently, we evaluated the performance of encapsulated human ASCs and proximal tubular cells (HK-2 cell line), elucidating the impact of these hydrogels on their viability, metabolic activity, proliferation, morphology, and renal phenotype. Our findings highlight the superior potential of dKECM hydrogels in promoting a sustained cellular activity and phenotype , underscoring their promise for CKD therapy. This study provides valuable insights into the potency of decellularized-based hydrogels as cell delivery vehicles, offering promising avenues for CKD treatment and kidney regeneration.

## Graphical abstract

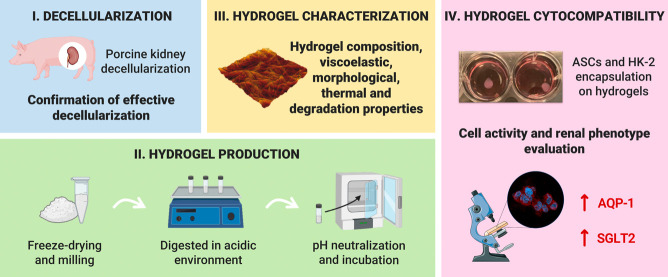

## 1 Introduction

Chronic kidney disease (CKD) represents a growing global health burden, fuelled by an aging population and the increasing prevalence of comorbid conditions like diabetes and hypertension. CKD affects >10% of the population worldwide,^1^ contributing to a substantial mortality rate (≈4.6% of global deaths),^2^ and it is projected to become the fifth leading cause of years of life lost by 2040.^3^ The hallmark of CKD is a progressive decline in kidney function, characterized by a cascade of structural and functional changes that comprises both functional renal cell loss and excessive deposition of extracellular matrix (ECM) components, predominantly collagen types I and III, and fibronectin.^4,5^ Untreated CKD ultimately progresses to end-stage renal disease (ESRD), for which renal replacement therapy (RRT) such as dialysis^6^ and kidney transplantation^7^ are the only available therapeutic options. While kidney transplantation offers a better long-term outcome, the organ shortage restricts its widespread application.^8^ Dialysis, while serving as a vital treatment option, has a set of complications that can significantly impact the health and well-being of patients,^6,9^ highlighting the urgent need for alternative and effective therapeutic strategies.

Advancements in stem cell-based therapies present promising outcomes in the treatment of several hard-to-treat diseases, like kidney injuries.^10^ Adipose-derived mesenchymal stem cells (ASCs) are an attractive option due to their easy isolation from adipose tissue harvested with minimal invasive methods.^11^ These stem cells possess the capacity for self-renewal and differentiation into various cell types, including adipocytes,^12^ osteoblasts,^13^ chondrocytes,^13^ and myocytes.^14^ Additionally, ASCs exhibit broad immunomodulatory properties, secreting a cocktail of diverse growth factors, cytokines, angiogenic factors, and anti-apoptotic molecules.^15,16^ Despite donor variations in proliferation rates, the differentiation capacity and properties of ASCs are preserved with aging.^17^ *In vitro* studies have shown that ECM components can guide stem cell differentiation toward specific lineages.^18–20^ For instance, previous reports confirmed the ability of renal ECM to promote the differentiation of human ASCs (hASCs) toward epithelial and tubular lineages.^21,22^

The local injection of stem cell suspensions provides more effective delivery to the target compared to systemic approaches, though challenges remain in cell homing, retention, and survival.^23,24^ To address these limitations, three-dimensional (3D) microenvironments like sponges,^25^ electrospun meshes,^26^ hydrogels,^21,27–32^ and organoids^31^ have been explored for improved cell delivery. In particular, injectable hydrogels, designed to mimic the native ECM, provide a versatile 3D platform with polymeric networks and highly hydrated environments.^27,29,33^ Although conventional hydrogel-forming materials, such as alginate^34^ and polycaprolactone (PCL),^35^ provide mechanical support for seeded cells, they lack important bioactive signals. Additionally, hydrogels derived from single ECM components, like collagen,^36^ heparin,^37^ hyaluronic acid (HA),^29^ or gelatin,^29,33^ still lack the full biochemical complexity of the kidney ECM milieu. Indeed, the native ECM environment is a complex network of cell-secreted products, providing both structural support and essential bioactive signals crucial for cells development and maintenance.^5,32^ Therefore, a growing interest in tissue- and organ-derived decellularized ECM (dECM) has developed, with methods for processing several tissues,^38,39^ including the kidney,^21,27,28,30,40^ into hydrogels for cell culture and injectable therapies. Previously our group demonstrated that decellularized kidney extracellular matrix (dKECM) retains native tissue proteins, glycosaminoglycans, and growth factors, while lacking immunogenic cellular components.^41^ More recently, to ensure the biocompatibility of dKECM hydrogels, we also confirmed their sterility and minimal immunogenic epitopes.^40^ *In vivo* experiments also showed that the dKECM hydrogel notably decreased the levels of oxidative stress and apoptosis, stimulating proliferation, secretion, and epithelial differentiation of ASCs.^21^ With Food and Drug Administration (FDA) approval and commercialization, decellularized matrices have already presented promising avenues in the field of regenerative medicine.^42,43^

Building upon previous research, this study aimed to investigate the potential of dKECM hydrogels as a platform for stem cell delivery and renal regeneration. To produce the dKECM hydrogels, decellularized porcine kidney tissue was used. First, we characterized the decellularized tissue samples to demonstrate the preservation of the ECM’s biophysical and biochemical properties. Subsequently, we compared the mechanical and structural properties of the produced dKECM hydrogels with a commercially available product consisting of a single ECM component, namely, a collagen I (Coll I) hydrogel. To elucidate their capacity to support cell culture while promoting or maintaining renal regeneration, two distinct cell types, human ASCs and HK-2, were encapsulated in the hydrogels. Through systematic examination of the interactions between the hydrogels and these cell types, we aimed to elucidate the impact of decellularized ECM on cellular behavior and differentiation toward kidney-specific phenotypes. This comparative analysis not only improves our understanding of the regenerative potential of dKECM hydrogels as a 3D niche for cell delivery but also provides valuable insights into optimizing their clinical translation for treating CKD.

## 2 Materials and Methods

### 2.1 Porcine Kidney Decellularization

Whole porcine kidneys were obtained from a local slaughterhouse and immediately stored at 4 °C until they could be further processed. The decellularization protocol, outlined in Figure 1, was adapted from a previous methodology developed by our group.^32^ Briefly, porcine kidneys were dissected into small pieces (∼7 mm × ∼7 mm × ∼5 mm). After being washed for 30 min, kidney samples were immersed in a solution of 1% sodium dodecyl sulfate (SDS) (catalog no. L3771, Sigma-Aldrich) and, then, in 1% Triton X-100 (catalog no. X100, Sigma-Aldrich) for 7 days (3.5 days on each solution) under agitation to facilitate cellular lysis. Subsequently, washes with phosphate-buffered saline (PBS, pH 7.4) (catalog no. P4417, Sigma-Aldrich) were performed for an additional 7 days to eliminate any residual detergent. These solutions were changed twice a day. To ensure complete removal of the cellular material, the decellularized tissue was treated overnight with 0.0025% (w/v) DNase I (catalog no. A3778, AppliChem) in a reaction buffer composed of 100 mM Tris hydrochloride (TrisHCl) (catalog no. T3253 Sigma-Aldrich), with 2.5 mM magnesium chloride (MgCl_2_) (catalog no. M2670, Sigma-Aldrich) and 0.5 mM calcium chloride (CaCl_2_) (catalog no. 102378 MERCK), at pH 7.5. Finally, the decellularized kidneys were immediately processed for analysis or frozen at −80 °C for further use.

**Figure 1:**
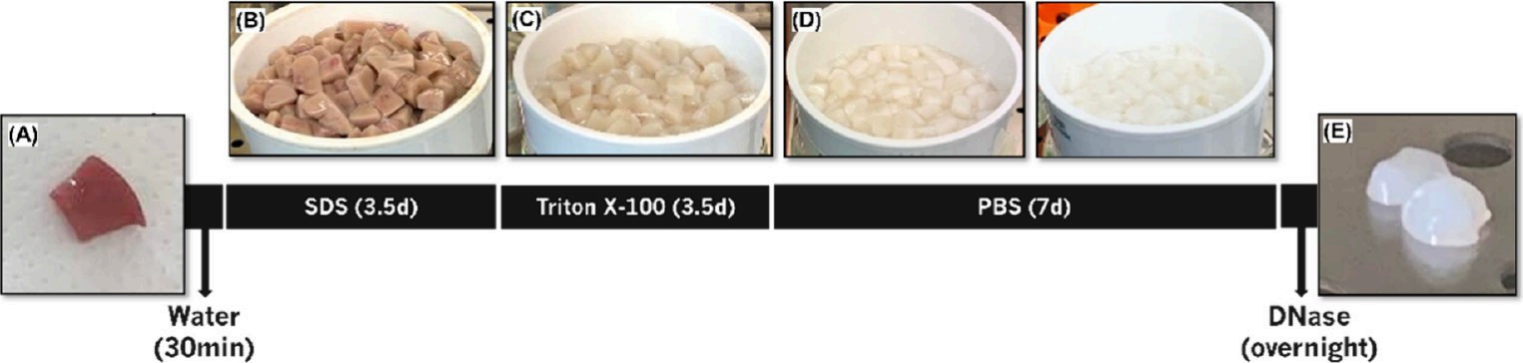
Representation of the decellularization process. (A) Native porcine kidney samples were obtained after sectioning. Then, they were immersed in water for 30 min, (B) treated with 1% SDS under agitation for 3.5 days, and (C) subsequently treated with 1% Triton X-100, under agitation, for an additional 3.5 days. (D) After treatment, samples were washed with PBS for 7 days. Then, the samples were immersed in 0.0025% (w/v) DNase I overnight. All solutions were changed twice a day. (E) Finally, decellularized porcine sections were obtained at the end.

### 2.2 Evaluation of the Effectiveness of the Decellularization Process

A comprehensive array of assays was implemented to evaluate the efficacy of the decellularization process applied to porcine kidney tissues. These methodologies targeted key parameters essential for successful decellularization, namely, the elimination of cellular remnants and the preservation of the native ECM.

#### 2.2.1 DNA Quantification

To quantify double-stranded DNA (dsDNA), before and after the decellularization process, the DNeasy blood and tissue kit (catalog no. 69504, QIAGEN) was used, following the manufacturer’s instructions.^32^ Sections from both native and decellularized kidney samples (25 mg) were digested for 3 h at 56 °C with proteinase K (catalog no. A3830, AppliChem). Then, the remaining DNA extraction was carried out using the provided spin column. Briefly, samples were lysed, followed by ethanol addition to facilitate the binding of DNA to the DNeasy silica membrane. Subsequent wash steps removed impurities, and finally, the purified DNA was eluted in a low-salt buffer provided by the kit, resulting in a concentrated DNA solution quantification. Finally, the dsDNA concentration (expressed as micrograms of DNA per milligram of wet tissue) was determined by measuring in triplicate the absorbance at 260 nm using the Nanodrop spectrophotometer (ThermoFisher Scientific).

#### 2.2.2 Histological Staining and Analysis

For histological analysis, native and decellularized kidney samples were first fixed in 10% formalin (catalog no. 5701, ThermoFisher Scientific) for 24 h. Then, they were embedded in paraffin and sectioned in 5 μm thick pieces. Histological slides were processed using an automatic stainer (HMS74, Microm). Hematoxylin and eosin (H&E, catalog nos. 7211 and 71204, respectively, ThermoFisher Scientific) and Masson Trichrome (MT, catalog no. 010210, Diapath) were used to assess the removal of nuclei and morphological ECM changes. While H&E staining was performed in the automatic stainer, the MT staining kit was used according to the manufacturer’s instructions. Following slide mounting, samples were observed in a Leica optical microscope (DM750, Leica) at magnifications of 10× and 40×.

#### 2.2.3 Assessment of the ECM Content

Toluidine blue O (TBO) staining was used to assess the quantity of proteoglycans (PGs) and glycosaminoglycans (GAGs) in both native and decellularized porcine kidney samples. Tissue samples (25 mg) were incubated in an aqueous solution of 15.24% TBO (1 mL, pH 10) (catalog no. 0300, Carl Roth) for 12 h under agitation at room temperature. Following incubation, the tissues were washed with a 0.1 mM sodium hydroxide (NaOH) (catalog no. 131687, Panreac Quimica) solution to remove unbound TBO molecules. Subsequently, 50% acetic acid (catalog no. 33209, Honeywell) was used to desorb the TBO molecules bound to the tissue for 10 min. The absorbance of the desorbed TBO solution was measured in triplicate at a wavelength of 633 nm by using a microplate reader (Synergy HT, Bio-Tek). To quantify the amount of TBO, a calibration curve was generated by measuring the absorbance of known TBO concentrations in 50% acetic acid at the same wavelength.

Sulfated PGs (sPGs) and GAGs (sGAGs) were quantified using the Blyscan sGAGs Assay (catalog no. B1000, Biocolor), according to the manufacturer’s protocol. To this end, sulfated PGs and GAGs were extracted from both native and decellularized kidney samples­(25 mg) using 0.01% papain (catalog no. P3125, Sigma-Aldrich) in 0.2 M sodium phosphate buffer (pH 6.4) at 65 °C for 12 h. Then, 50 μL of each supernatant was transferred to a new tube, and 1 mL of the dye reagent was added. The resulting mixture was gently mixed and incubated at room temperature for 30 min. After another centrifugation, the supernatant was discarded, the dissociation reagent added to the pellet, and the mixture incubated for 10 min. The absorbance of the samples was measured in triplicate at a wavelength of 656 nm using a microplate reader (Synergy HT, Bio-Tek). Finally, the content of sulfated PGs and GAGs was calculated using a standard curve.

### 2.3 dKECM Processing and Hydrogel Preparation

Frozen decellularized kidney samples underwent a 48 h freeze-dryer cycle (LyoAlfa 10/15, Telstar). To obtain a fine powder, lyophilized samples were processed with a cryogenic grinder (SPEX SamplePrep). The powder was stored at −20 °C until further use. A concentration of 2% was selected with the aim of balancing mechanical stability and bioactivity, drawing upon results from prior research conducted by our group.^32^ To produce hydrogels, 2 mg/mL dKECM powder was first digested with 0.2 mg/mL pepsin (catalog no. 10264440, Fisher Scientific) in 0.01 M hydrochloric acid (HCl) (catalog no. 30721, Honeywell) for 48 h. All solutions were filtered through a 0.22 μm filter before use. After digestion, the pregel solution can be stored at −20 °C for short-term usage. To finalize the preparation of the hydrogels, the pH of the pregel solution was adjusted to physiological pH by adding 0.1 N NaOH, and the osmotic pressure was regulated by adding a 1/9 ratio of 10× PBS, on ice. Coll I hydrogels were prepared similarly. Briefly, the commercially obtained Coll I solution from rat tail (catalog no. 08-115, Sigma-Aldrich) was diluted to a concentration of 2 mg/mL using 10× PBS and adjusted to the desired concentration with culture medium. The pH of this pregel was also adjusted to 7.4 using 0.1 M NaOH. After neutralization, the pregel solutions were immediately used. To ensure uniform hydrogel samples, 100 μL of each gel type was pipetted into silicone molds and incubated for 45 min at 37 °C.

### 2.4 dKECM and Coll I Hydrogel Characterization

dKECM hydrogels, rich in various ECM components, were extensively characterized for their structure and composition to evaluate their potential as cell delivery vehicles compared to Coll I hydrogels. Key assessments included the gelation temperature, degradation rate, rheology, ECM composition, protein structure, thermal transitions, and surface topography.

#### 2.4.1 Assessment of the Composition of Hydrogels

To compare the components present in both 2% dKECM and Coll I hydrogels, we employed the same methodology described in section 2.2.3 (ECM content assessment). A 100 μL portion of each hydrogel was analyzed.

#### 2.4.2 Vial Inversion Test

The gelation capability of 2% dKECM and Coll I hydrogels was determined using the vial inversion assay.^44^ Aliquots of 200 μL of the hydrogel were pipetted into vials and subjected to a range of temperatures (5, 15, 25, and 37 °C) using a Thermomixer (Eppendorf). At each temperature, the vials were inverted, and the gelation state was evaluated on the basis of the hydrogel’s ability not to flow. The gelation temperature was defined as the temperature at which the hydrogel underwent the transition from a liquid to a gel state, indicated by increased resistance to flow during vial inversion.^45^

#### 2.4.3 Rheological Characterization

To understand the injectability and subsequent gelation behavior of the hydrogels, the viscoelastic properties of the 2% dKECM and Coll I hydrogels were evaluated using a Kinexus Pro Rheometer (Malvern Instruments). To this end, a parallel plate geometry with a 20 mm diameter and a 1 mm gap was employed for all measurements. A volume of 320 μL of each solution was pipetted onto the plate, with water around as a solvent trap. After incubation at 37 °C for 45 min for solution gelation, the linear viscoelastic (LVE) region was determined at a constant frequency of 1 Hz by strain sweep tests. Subsequently, frequency sweep tests were conducted, subjecting the hydrogels to a constant strain of 1% while the frequency ranged from 1 to 100 rad/s. Following each test, a new sample was utilized. Temperature sweep analysis is crucial for temperature-sensitive samples, providing valuable insights into gelation kinetics before and after gelation. To this end, solutions underwent a constant oscillatory stress of 1% at a frequency of 1 Hz, with the temperature increasing from 4 to 37 °C at a rate of 2 °C/min. Finally, the viscosity of both 2% dKECM and Coll I hydrogels was assessed using viscometry tests, which involved a shear rate sweep from 0.01 to 500 s^–1^ for 2 min at two different temperatures, namely, 5 °C (optimal pregel temperature) and 25 °C (room temperature). Viscometry tests measured the resistance to flow under varying shear forces, which is essential for the optimization of injectable hydrogels. This step ensured appropriate viscosity profiles for efficient delivery and further gelation, crucial for targeting injuries in the kidney.^32^

#### 2.4.4 Circular Dichroism (CD)

To analyze the secondary structure of the dKECM and Coll I, before and after gelation, CD spectroscopy was employed. A small volume of each solution was pipetted into a dismantled cuvette (catalog no. 106-0.01-40, Hellma), with a path length of 0.01 mm. A CD spectrometer (catalog no. J1500, Jasco) was used to acquire CD spectra between 180 and 260 nm with a bandwidth of 1 nm. Each spectrum was obtained by averaging three individual measurements. Additionally, to ensure accurate analysis of protein secondary structure, control samples lacking dKECM particles or Coll I were used as baselines for their respective hydrogel counterparts.

#### 2.4.5 Differential Scanning Calorimetry (DSC)

The thermal properties of 2% dKECM and Coll I hydrogels were measured using a differential scanning calorimeter (Q100, TA Instruments). To prevent water evaporation, the samples were frozen at −80 °C and then freeze-dried (LYOQUEST −85 °C PLUS ECO, Telstar) overnight. Approximately 2 mg of each freeze-dried sample was placed in an aluminum pan. The samples were then heated from 0 to 120 °C at a controlled rate of 2 °C/min, under a continuous nitrogen purge (50 mL/h) to maintain an inert atmosphere within the instrument chamber.

#### 2.4.6 Atomic Force Microscopy (AFM)

To assess the surface topography of 2% dKECM and Coll I hydrogels, AFM analysis was employed. Briefly, 10 μL of each solution was pipetted over glass slides and incubated at 37 °C for 15 min, to allow for complete gelation. Following incubation, the samples were air-dried for 2 h. AFM imaging was performed at room temperature by using a JPK Nanowizard 3 instrument. The AC mode with ACTA probes (*k* ∼ 37 N/m) was employed to analyze the hydrogel’s morphological features. The probe had a drive frequency of ∼254 kHz and a scanning speed of 1.0 Hz. Images were acquired at a resolution of 512 pixels × 512 pixels and analyzed using the JPK data processing software.

#### 2.4.7 Scanning Electron Microscopy (SEM)

The general morphology of both hydrogels was evaluated by using SEM. Initially, all samples were fixed in 2.5% glutaraldehyde (catalog no. G5882, Sigma-Aldrich), washed twice with PBS (5 min each time), and then dehydrated using a series of graded ethanol concentrations (ranging from 20% to 100%, 20 min incubation for each). After dehydration, the samples were dried with a critical point dryer system (Autosamdri-815, Tousimis) in a 45 min cycle and subsequently coated with gold via sputter coating (108A, Cressington). The samples were then examined by using a SEM instrument (JSM-6010 LV, JEOL) at an acceleration voltage of 10 kV.

#### 2.4.8 Weight Loss and Water Uptake

To determine the weight loss, 2% dKECM and Coll I hydrogel samples were initially weighted (*W*_initial_) and then immersed in 0.5 mL of an isotonic solution of 0.154 M sodium chloride (NaCl) (catalog no. 31434, Sigma-Aldrich) at pH 7.4. Subsequently, hydrogels were incubated at 37 °C. At predefined time points (1, 3, 7, 14, and 21 days), the surrounding solution was removed, and a filter paper was used to absorb excess water. Then, the weight (*W*_final_) of the resulting hydrogels was measured. The percentage of weight loss was determined using eq 1:

$$\mathrm{weight loss (\%)}=\left(\frac{W_{\mathrm{initial}}-W_{\mathrm{final}}}{W_{\mathrm{initial}}}\right)\times 100$$ 1

To assess the water uptake potential of the hydrogels over the same period, samples were weighed (*W*_wet_), frozen at −80 °C, and then freeze-dried (LYOQUEST −85 °C PLUS ECO, Telstar). Subsequently, the dried samples were weighed (*W*_dry_). The water uptake percentage was calculated using eq 2:

$$\mathrm{water uptake (\%)}=\left(\frac{W_{\mathrm{dry}}-W_{\mathrm{wet}}}{W_{\mathrm{wet}}}\right)\times 100$$ 2

### 2.5 Cell Culture and Encapsulation within the Hydrogels

Two cell types, hASCs and HK-2, were employed to assess the biological potential of 2% dKECM and Coll I hydrogels. The hASCs were isolated from adipose tissue samples obtained from the abdominal region of healthy donors undergoing liposuction surgery. These samples were obtained from donors at the Hospital da Prelada (Porto, Portugal) with informed consent. Isolation of hASCs from the lipoaspirates employed enzymatic digestion, following a previously established protocol.^46^ The hASCs were cultured in α minimum essential medium (α-MEM) (catalog no. 12000063, Gibco, ThermoFisher Scientific) supplemented with 10% (v/v) fetal bovine serum (FBS) (catalog no. A3160802, Gibco, ThermoFisher Scientific) and 1% (v/v) antibiotic/antimycotic solution (catalog no. 15240062, Gibco, ThermoFisher Scientific). Additionally, human kidney cortex/proximal tubule cells (HK-2 cell line, ATCC CRL-2190, ATCC) were also used as the control cell type. HK-2 cells were expanded in keratinocyte serum-free medium (catalog no. 17005042, Gibco, Thermo-Fisher Scientific) supplemented with 0.05 mg/mL bovine pituitary extract (BPE) and 5 ng/mL epidermal growth factor (EGF). Cell cultures were maintained at 37 °C in a 5% CO_2_ high-humidity environment. The culture medium was renewed every 2–3 days, and cells were subcultured at 80% confluence until sufficient numbers of cells were attained to initiate the experiments.

Prior to cell encapsulation, pregel solutions were thawed at 4 °C for 2 h. In the meantime, the cultured cells were detached for further encapsulation. Briefly, adherent cells were washed with Dulbecco’s phosphate-buffered saline (DPBS) (catalog no. 21600044, Gibco, ThermoFisher Scientific) and incubated with TrypLE Express (catalog no. 12605028, Gibco, ThermoFisher Scientific) for 5 min at 37 °C for their detachment. Then, cells were collected, centrifuged, and resuspended in a minimal volume of culture medium. After hydrogel neutralization to physiological pH, as previously described, 500 000 cells/mL were resuspended in this solution. The pregel with cells was then pipetted into 100 μL molds and incubated at 37 °C in 5% CO_2_ for ≥2 h for complete gelation. Subsequently, the cell-laden hydrogels were transferred to a 48-well plate and cultured with the respective medium over a period of 21 days. The culture medium was replaced every 2–3 days.

### 2.6 Evaluation of the Cellular Response

The porcine-derived dKECM hydrogels developed in this study were designed for future integration into the biological milieu. Therefore, validating their cytocompatibility and assessing their ability to support existing renal phenotypes or to induce renal differentiation are crucial. hASCs and HK-2 cells were encapsulated with in 2% dKECM or Coll I hydrogels, and assays over 21 days evaluated cell viability, metabolic activity, proliferation, morphology, and renal-specific phenotypes to understand the cell–material interactions for renal therapy applications.

#### 2.6.1 Cells Viability Imaging

The live/dead assay was employed to assess the viability of hASCs and HK-2 cells encapsulated with in the hydrogels. At days 1, 7, and 21, hydrogels were transferred to a new well plate and rinsed with sterile DPBS. Then, 500 μL of each medium with 2 μg/mL Calcein-AM (catalog no. 80011-2, VWR) and 1 μg/mL propidium iodide (PI) (catalog no. P1304MP, Invitrogen, ThermoFisher Scientific) was added to each sample. After being incubated for 30 min, samples were washed three times with DPBS and promptly visualized in the confocal microscope (LSM 980, Zeiss).

#### 2.6.2 Cells Metabolic Activity

The metabolic activity of cells encapsulated within the hydrogels was assessed at the mentioned time points using the AlamarBlue assay (catalog no. 424702, BioLegen). Briefly, the hydrogels were washed twice with DPBS and transferred to a new well plate. Subsequently, fresh culture medium supplemented with 10% (v/v) AlamarBlue reagent was added to each sample. Following a 4 h incubation period, the supernatant was collected for fluorescence intensity measurement using a microplate reader (Synergy HT, Bio-Tek). Excitation and emission wavelengths were set at 530/25 and 590/25 nm, respectively.

#### 2.6.3 Cells Proliferation

The DNA content was quantified to access the cells proliferation rate over a 21 day culture. Briefly, samples for each condition and time point were subjected to an overnight digestion with proteinase K in a buffer containing a 100 mM Tris solution (catalog no. T3253 Sigma-Aldrich), 5 mM EDTA (catalog no. E5134, Sigma-Aldrich), 200 mM NaCl, and 0.2% (v/v) SDS at 37 °C under shaking. Following digestion, samples were centrifuged, and the supernatant was collected for DNA isolation. DNA was then precipitated using 2-propanol (catalog no. 327272500, ThermoFisher Scientific) and further purified by being washed with cold 70% ethanol (catalog no. LB0484, AppliChem). DNA pellets, after being air-dried for 30 min, were resuspended in 100 μL of an 8 mM NaOH solution and incubated for 1 h at room temperature. Then, DNA extracts were stored at −80 °C for subsequent quantification. DNA quantification was conducted using a Nanodrop spectrophotometer (ThermoFisher Scientific).

#### 2.6.4 Cells Morphology and Location within the Hydrogels

To assess the cell distribution and morphology within the hydrogels over time, histological staining was performed on days 1, 7, and 21. Briefly, fixated hydrogels were sectioned, and two specific stains were employed. The staining procedure was conducted as detailed in section 2.2.2 (histological analysis of decellularized and native kidney).

#### 2.6.5 Evaluation of Cells Phenotype Expression

The ability of dKECM and Coll I hydrogels to modulate the phenotype of hASCs and HK-2 cells was assessed using immunocytochemistry (ICC) analyses on days 1, 7, and 21. First, the hydrogels were fixed overnight at 4 °C with 10% (v/v) neutral buffered formalin. After PBS washing, the samples were embedded in paraffin and sectioned into 5 μm thick slices. After deparaffinization, antigen retrieval was performed in citrate buffer (catalog no. A11156, Alfa Aesar) containing 0.05% (v/v) Tween 20 (catalog no. P1379, Sigma-Aldrich), using a microwave Cook n grill (Sanyo) for 4 min (15 s on, 15 s off). Following cooling, sections were permeabilized with 0.2% (v/v) Triton X-100 in PBS and then incubated with 3% (w/v) bovine albumin serum (BSA) (catalog no. A2153, Sigma-Aldrich), to minimize nonspecific binding. Primary antibodies (listed in Table 1) to target kidney markers of interest were diluted in a 1% (v/v) blocking solution and incubated with the hydrogel sections overnight at 4 °C. After being washed with PBS containing 0.05% (v/v) Tween 20, sections were incubated with the corresponding secondary antibody for 1 h at room temperature. In this step, cell nuclei were also stained with DAPI (1:1000). Finally, stained sections were washed, mounted, and visualized using a Leica confocal microscope (LSM 980, Zeiss).

**Table 1:** Primary and Secondary Antibodies Used for the ICC Analysis

primary antibody	dilution ratio	secondary antibody	dilution ratio
CD133	1:200	Alexa Fluor 594 donkey anti-mouse IgG	1:1000
Wilms’ tumor protein (WT1)	1:20		
paired box gene 2 (PAX2)	1:100	Alexa Fluor 594 donkey anti-rabbit IgG	1:1000
nephrin (NPHS1)	1:200		
podocin (NPHS2)	1:65		
aquaporin-1 (AQP-1)	1:100		
sodium-dependent glucose transporter 2 (SGLT2)	1:30		

## 3 Statistical Analysis

All quantitative data are expressed as the mean ± standard deviation (SD) of at least three independent experiments. GraphPad Prism (version 8.01, GraphPad Software, San Diego, CA) was used to determine statistical differences. The normality of the data was assessed using the Shapiro–Wilk test. For normally distributed data, parametric tests were employed. Two-tailed Student’s *t* tests were used to compare two groups. For data exhibiting a non-normal distribution, nonparametric tests were employed. A two-way analysis of variance on ranks test followed by Sidak’s multiple-comparison test was used to assess differences between groups. A *p* < 0.05 significance level was considered for all statistical tests.

## 4 Results and Discussion

### 4.1 Confirmation of Effective Decellularization

For porcine kidney decellularization, anionic (SDS) and non-ionic (Triton X-100) detergents were used to eliminate residual nucleic acids. To corroborate the efficacy of the decellularization protocol, a qualitative and quantitative comparative analysis between native and decellularized kidney tissues was conducted. This analysis provided data supporting the suitability of the decellularized matrix for subsequent use.

The resulting decellularized kidney tissues exhibited a dsDNA content (18.95 ng/mg) significantly lower than that of the native tissue (865.1 ng/mg) (Figure 2A) This value is far below the established threshold for adequate decellularization (50 ng/mg of dry weight),^47^ representing a 97.8% reduction in dsDNA content. Histology analysis of the tissues (Figure 2B–E) was also performed, aiming to support the quantitative findings and to confirm the preservation of the ECM. Accordingly, H&E staining (Figure 2B,C) confirmed the successful removal of nucleic material. Additionally, MT staining (Figure 2D,E) demonstrated complete removal of cytoplasmic residues while preserving the collagenous structure within the decellularized tissue.

**Figure 2:**
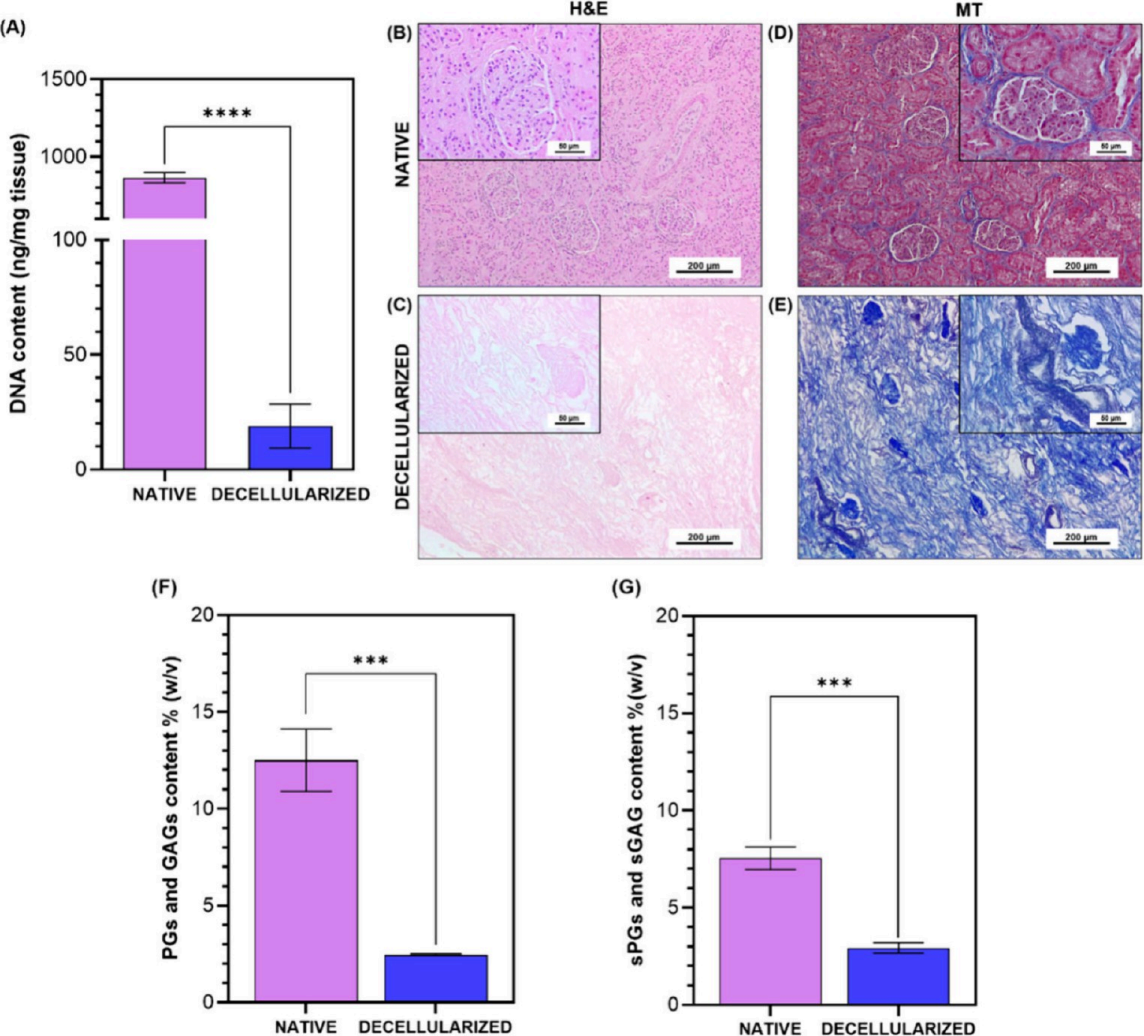
Decellularization of porcine kidney tissues. (A) dsDNA quantification in native and decellularized porcine kidney tissues. Statistically significant difference between native and decellularized tissues (****p < 0.0001). Histological images of kidney tissues. Hematoxylin and eosin (H&E) and Masson’s Trichrome (MT) staining of native (B and D, respectively) and decellularized kidney (C and E, respectively) tissues. The scale bars are 200 and 50 μm. (F) Quantification of the total content of soluble PGs and GAGs in native and decellularized kidney tissues. Statistically significant difference between native and decellularized tissues (***p < 0.001). (G) Quantification of soluble sPGs and sGAG in native and decellularized kidney. Statistically significant difference between native and decellularized tissues (***p < 0.001).

Preserving the ECM structure, including proteins like collagen, fibronectin, laminin, and GAGs, is critical not only to influence cell behavior but also to enhance effective hydrogel formation.^48^ Therefore, the composition of PGs and GAGs was quantified in the native and decellularized kidney tissues (Figure 2F). The content of PGs and GAGs before decellularization was 12.52 ± 1.62% (w/v), decreasing to 2.47 ± 0.04% (w/v) after decellularization. Although statistically significant differences (*p* = 0.0002) were observed in the overall content between native and decellularized tissues, ∼20% of the native ECM content was retained in the decellularization process. The sPGs and sGAGs, essential ECM components for maintaining tissue integrity and function, were also quantified (Figure 2G). The concentrations of sPGs and sGAGs were 7.54 ± 0.58% (w/v) before decellularization and 2.94 ± 0.27% (w/v) following this process. This revealed that decellularized tissues retained ∼39% of the native content of sPGs and sGAGs. Altogether, these observations are aligned with previous results obtained by our group.^41^ The decellularization protocol effectively removed dsDNA, a crucial step to ensure immune tolerance, while preserving essential components, namely, collagen and GAG content, within the decellularized ECM. These findings suggest a successful production of an acellular ECM suitable for further investigation.

### 4.2 Characterization of the Hydrogel

Hydrogels are known for creating a 3D microenvironment that supports and guides cell behavior and differentiation. Assessing the intrinsic properties of dKECM hydrogels is crucial as their physicochemical characteristics impact cellular processes.

#### 4.2.1 Assessment of the Hydrogel Composition

As expected, a significant difference in the composition of key ECM biomolecules between 2% dKECM and Coll I hydrogels was observed (Figure 3). The dKECM hydrogels exhibited a notably higher abundance of two crucial components, PGs and GAGs (Figure 3A). Indeed, dKECM hydrogels possessed a significantly higher content [9.5 ± 1.9% (w/v)] compared to that of Coll I hydrogels [3.0 ± 0.30% (w/v)]. A more prominent presence of these sulfated components of the ECM was also observed (Figure 3B), which are fundamental for cell adhesion, migration, and differentiation. The dKECM hydrogel exhibited soluble ECM composition values near those found in native kidney tissue, corroborating an effective decellularization process.

**Figure 3:**
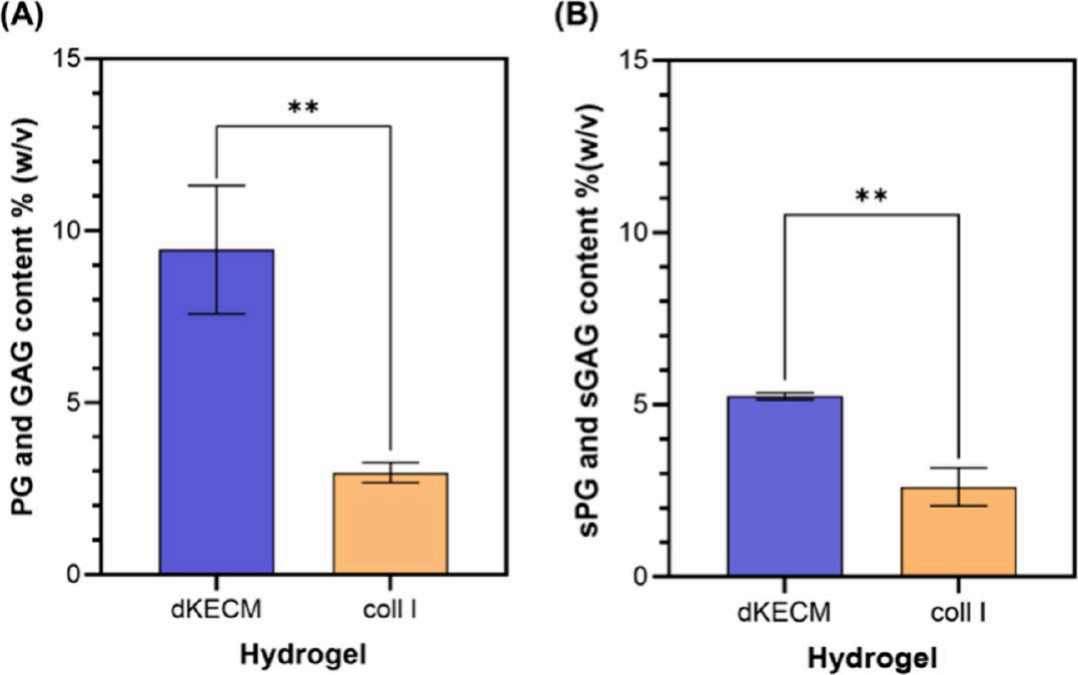
Assessment of the hydrogel composition. (A) Overall content of soluble PGs and GAGs of 2% dKECM and Coll I hydrogels. Statistical difference between dKECM and Coll I hydrogels (**p < 0.01). (B) Soluble sGAG and sPG quantification of 2% dKECM and Coll I hydrogels. Statistical difference between dKECM and Coll I hydrogels (**p < 0.01).

#### 4.2.2 Thermal Gelation

Vial inversion testing confirmed the successful gelation of 2% dKECM (Figure 4A) and Coll I (Figure 4B) hydrogels at 37 °C. Notably, some transitioning to a gel-like state was already observed at 25 °C. The thermosensitive properties of the hydrogels were confirmed, and the Coll I hydrogel displayed a visible color shift from transparent to opaque upon gelation.

**Figure 4:**
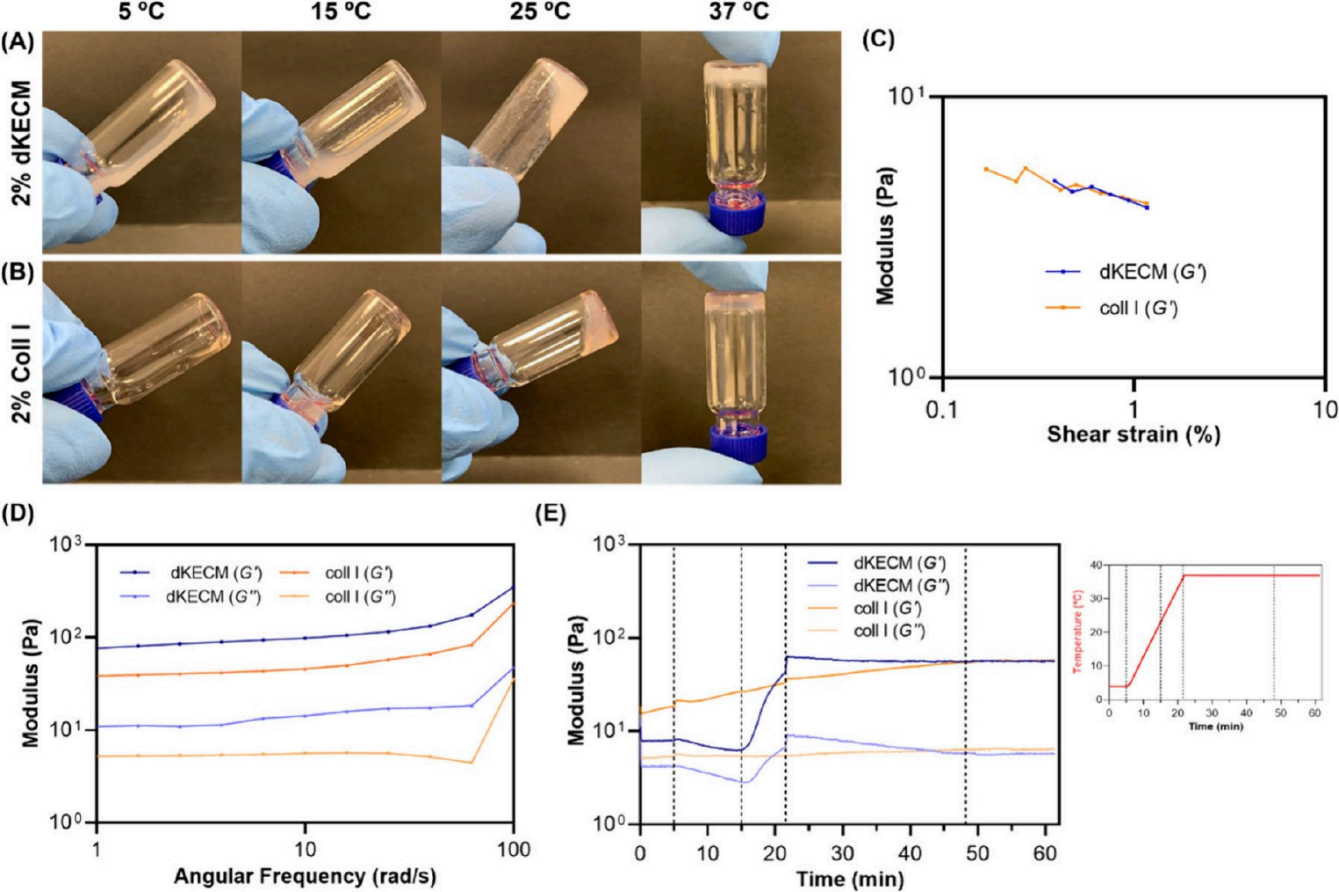
Hydrogel gelation behavior. Vial inversion testing of (A) 2% dKECM and (B) Coll I hydrogels at 5, 15, and 25 °C and after successful gelation at 37 °C. (C–E) Rheological characterization of 2% dKECM and Coll I hydrogels. (C) Strain sweep results, showing the linear viscoelastic region (LVR) values in terms of shear strain (percent) and respective *G′* (Pascal), at an oscillatory shear stress frequency of 1 Hz. (D) Frequency sweep tests at a strain of 1% with frequencies ranging from 1 to 100 rad/s. The storage modulus (*G′*) and loss modulus (*G′′*) were obtained after gelation. (E) Time sweep test on hydrogels at a constant oscillatory stress of 1% and a frequency of 1 Hz, with a temperature ramp after 5 min from 4 to 37 °C at a rate of increase of 2 °C/min. The gelation kinetics of both hydrogels were observed over 1 h.

#### 4.2.3 Rheological Characterization

Rheological analysis evaluated the viscoelastic properties and stability of 2% dKECM and Coll I hydrogels, essential for their use as injectable cell delivery systems. Both hydrogels exhibited a measurable LVE region, determined by strain sweep tests (Figure 4C), with no statistically significant differences observed (*p* > 0.05). dKECM hydrogels exhibited an LVE strain (1.30 ± 0.05%) slightly larger than that of the Coll I hydrogels (1.11 ± 0.01%) but close to 1%. Therefore, a strain of 1% was selected for subsequent tests of both hydrogels. Frequency sweep tests (Figure 4D) revealed that both hydrogels exhibited gel-like behavior, with the storage modulus (*G*′) being consistently higher than the loss modulus (*G′′*) across the range of 0.01–100 Hz.^32^ Moreover, the *G′* of dKECM hydrogels was higher than that of Coll I hydrogels, suggesting a more robust and elastic network likely due to the natural ECM components in its composition (e.g., collagens, laminins, and proteoglycans) that contribute to a stronger intermolecular self-assembly.^50^ The measured storage moduli of the dKECM (127 ± 41.8 Pa) and Coll I (67.4 ± 6.25 Pa) hydrogels also fall within the expected range of values observed for the native porcine kidney tissue (30–500 Pa),^51^ suggesting a successful replication of the mechanical properties. Moreover, the strength of the hydrogel matrix significantly influences cellular activity, with mechanical and biological cues from the native kidney ECM playing essential roles. Matrix stiffness impacts key processes, such as adhesion, migration, proliferation, and differentiation. On very soft matrices, weaker cell adhesion hinders migration while overly stiff matrices restrict migration, limiting the cells’ ability to move and reorganize within the matrix. Additionally, the matrix stiffness plays a critical role in regulating differentiation. For example, mesenchymal stem cells (MSCs) cultured on matrices mimicking different tissue types, such as brain (0.1–1 kPa), muscle (8–17 kPa), and bone (25–40 kPa), differentiated into neurogenic, myogenic, and osteogenic lineages, respectively.^49^

The influence of temperature on hydrogel gelation kinetics was assessed using time sweep tests (Figure 4E). In 2% dKECM hydrogels, *G′* values slightly decreased from 4 to 25 °C, followed by a significant increase at 37 °C (62.4 Pa), with gelation occurring after 16.6 min. In contrast, 2% Coll I hydrogels showed a nearly linear increase in *G′*, from 4 to 25 °C Pa, reaching a peak at 37 °C (36.8 Pa) after 44.5 min. Despite these differences, both hydrogels reached similar equilibrium *G′* values after 44.5 min: dKECM (56.3 Pa) and Coll I (57.0 Pa). Notably, both hydrogels showed increasing *G′* values before reaching 37 °C, with collagen fibril assembly occurring sharply after 25 °C for dKECM and gradually from 4 °C for Coll I. These data quantitatively support the observations previously described for the vial inversion test. Shear rate sweep tests demonstrated that both hydrogels exhibited shear-thinning behavior, which is essential for injectable biomaterials,^52^ but dKECM hydrogels demonstrated a more significant viscosity decrease compared to Coll I (Figure S1).

#### 4.2.4 Conformational Changes and Thermal Properties

CD spectroscopy was used to investigate the conformational changes in dKECM and Coll I hydrogels during gelation (Figure 5A). The 2% dKECM hydrogels exhibited a prominent negative peak at ∼200 nm in both pre- and post-gelation states indicative of α-helical and random coil conformations.^53^ The positive peak at ∼225 nm suggests β-sheet conformations,^53^ bieng representative of fibronectin^54^ and laminin.^55^ Both hydrogels showed a negative peak from 230 to 250 nm indicative of GAGs.^56^ The CD spectra of 2% dKECM hydrogels remained similar after gelation, suggesting minimal structural changes and a consistent profile of a protein mixture.^41^ Conversely, 2% Coll I hydrogels displayed a significant phase transition upon gelation with a new spectral profile emerging, characteristic of triple-helix formation in Coll I.^57^

**Figure 5:**
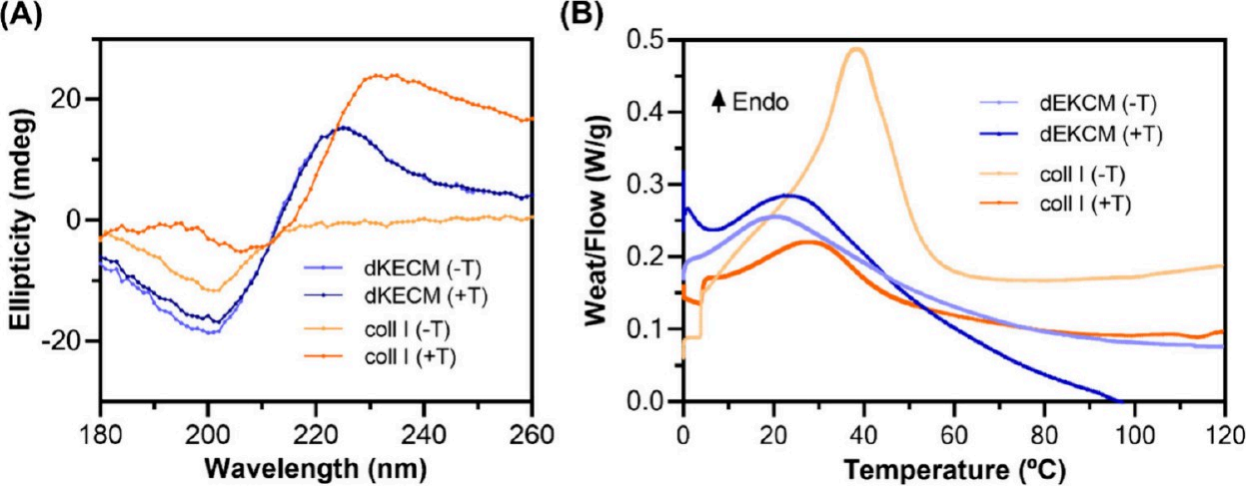
Structural and thermal properties of 2% dKECM and Coll I hydrogels, before (−T) and after (+T) gelation. (A) CD spectra and (B) DSC thermograms of both hydrogels.

DSC thermograms (Figure 5B) for 2% dKECM and Coll I hydrogels demonstrated a single endothermic peak associated with a phase transition. For 2% dKECM hydrogels, the temperature and energy of this transition remained consistent before (24.19 °C and 65.73 J/g, respectively) and after gelation (26.42 °C and 67.56 J/g, respectively), suggesting minimal thermal alteration consistent with the CD spectra. Conversely, 2% Coll I hydrogels displayed a significant decrease in temperature and energy for this transition before and after gelation (36.94 to 24.67 °C and 112.40 to 54.71 J/g, respectively), indicating structural reorganization and likely coll denaturation at ∼40 °C.^58^ This thermal shift also coincides with the observed changes in the CD spectra of the 2% Coll I hydrogel. Indeed, the collagen molecules will undergo a transition from a less stable, random coil conformation (before gelation) to a more stable, triple-helical structure (after gelation).^59,60^

#### 4.2.5 Morphological Characterization

SEM micrographs (Figure 6A,B) revealed a shared feature between 2% dKECM and Coll I hydrogels, namely loosely arranged interconnected fibers in a porous network, resembling a collagenous matrix. However, dKECM hydrogels also displayed additional mesh-like zones, indicating the presence of other biomolecules, such as PGs and GAGs.^50^ AFM analysis (Figure 6C–F) confirmed the fibrillar nature of both hydrogels. Coll I hydrogels exhibited well-defined, smooth, and aligned fibrils, resembling intertwined ropes (Figure 6D,F). In contrast, dKECM hydrogels displayed a more intricate and heterogeneous surface topography with noticeable irregularities, texture variations, and reticular regions (Figure 6C,E). Topographic analysis revealed fine singular fibers in dKECM hydrogels, ranging from 20 to 100 nm, forming larger networks with diameters between 100 and 150 nm. In contrast, Coll I hydrogels had thicker individual fibers (100–150 nm), creating larger networks (300–500 nm). SEM and AFM analyses highlighted distinct nano/microscale morphologies between the two hydrogels, likely reflecting their different molecular compositions.

**Figure 6:**
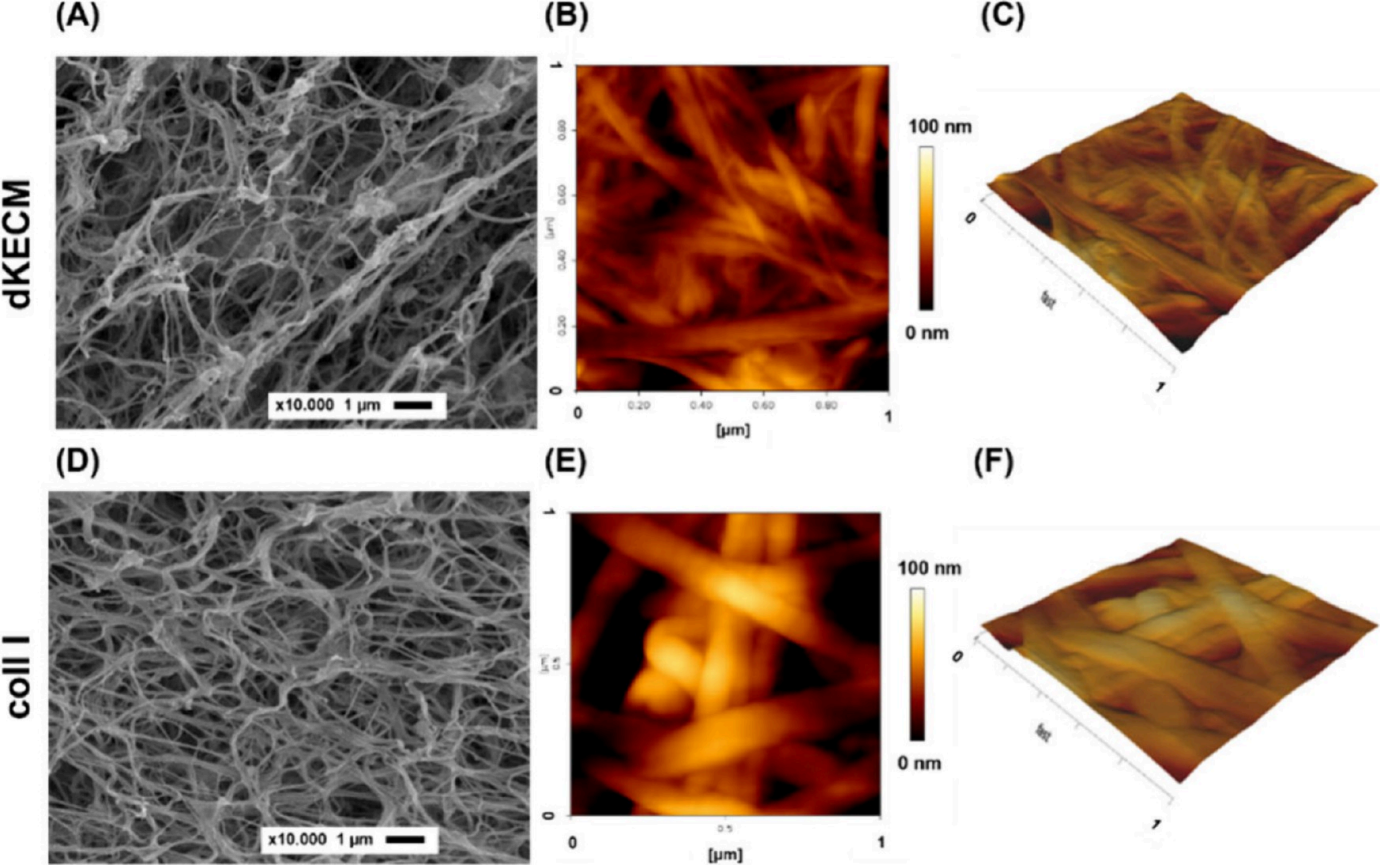
Morphological and topographical characterization of 2% dKECM and Coll I hydrogels. (A and D) SEM micrographs. The scale bars are 5 μm (up) and 1 μm (down). (B, C, E, and F) AFM micrographs [scales of 10 μm (top) and 1 μm (bottom)]: (B) 2% dKECM hydrogel topography and (C) the respective 3D representation, and (E) 2% Coll I hydrogel and (F) the respective 3D representation.

#### 4.2.6 Weight Loss and Water Uptake

The stability of 2% dKECM and Coll I hydrogels was assessed by weight loss and water uptake measurements over 21 days. The Coll I hydrogels after the first day exhibited a significantly larger weight loss (6.7%) compared to that of the dKECM hydrogels (1.2%) (Figure 7A). This suggests a faster release of readily soluble components within the Coll I hydrogel. Moreover, the dKECM hydrogels displayed a slower and more gradual weight loss profile throughout the timeframe. Indeed, after 21 days, dKECM hydrogels exhibited significantly greater mass retention (∼91.4%) compared to that of Coll I hydrogels (∼86.2%). These findings are consistent with prior studies indicating that incorporating dECM (in particles or solution) into Coll I scaffolds reduces the kinetics of mass loss. With regard to the water absorption capacity (Figure 7B), both hydrogels exhibited a similar water uptake profile during the 21 days. Interestingly, the Coll I hydrogels displayed a higher initial water uptake (∼6.9%) compared to that of the dKECM hydrogels (∼4.5%). This might be due to the more open structure of the Coll I matrix compared to the potentially denser dKECM network. However, these differences became less evident over time. Indeed, dKECM hydrogels showed water absorption slightly higher than that of Coll I hydrogels after 3 and 7 days. Overall, the controlled degradation profiles of dKECM hydrogels suggest a prolonged functionality and better support for cell delivery compared to those of Coll I hydrogels. Swelling and degradation of dKECM-based hydrogels are strongly influenced by the ECM content, temperature, and pH due to their effects on collagen fibrillogenesis and the overall structural integrity of the 3D network.^59^ At physiological pH (7.4), achieved by neutralizing an acidic dKECM pregel solution with NaOH, collagen forms stable fibrillar structures, as previously demonstrated in Figure 6. Deviations from this pH have been demonstrated to disrupt the balance of electrostatic interactions on collagen hydrogels, impacting their water uptake behavior.^61^ It has been demonstrated that, for Coll I hydrogels at acidic pH (2.5), collagen remains soluble despite increasing salt concentrations, while at neutral pH (7.0), D-banded fibrils form at intermediate NaCl concentrations. Additionally, fibrillogenesis is less efficient at a higher pH (∼9.0).^62^ The neutralization process must also be performed on ice to control ECM content polymerization, which occurs at a physiological temperature (37 °C).^40,59,63^ For instance, for porcine myocardial tissue ECM hydrogels, reducing the gelation temperature below 22 °C was found to inhibit gelation, in contrast to pure collagen hydrogels, which are capable of gelling between 4 and 37 °C.^64^ By its side, higher temperatures can denature ECM components,^58^ compromising hydrogel stability and accelerating degradation.

**Figure 7:**
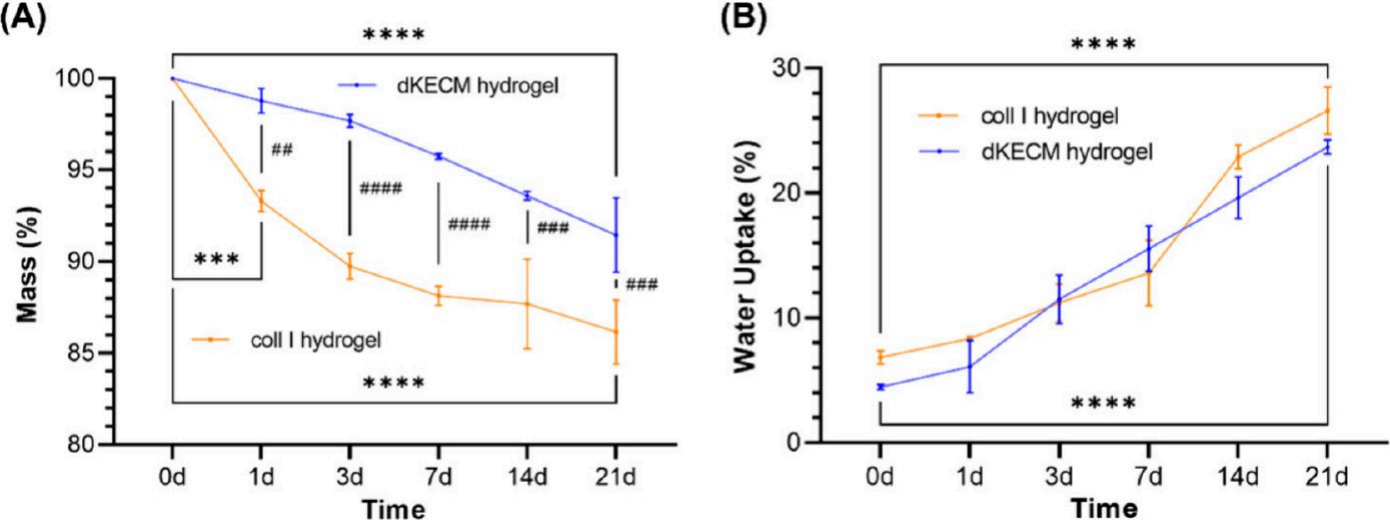
Weight loss and water uptake profile of 2% dKECM and Coll I hydrogels over 21 days. (A) Weight loss ratio of dKECM and Coll I hydrogels. Statistical difference between 2% Coll I at 0 days vs 1 day (***p < 0.001), 2% dKECM and Coll I hydrogels at 0 days vs 21 days (****p < 0.0001), 2% dKECM vs Coll I hydrogels (1 day) (^##^ *p* < 0.01), 2% dKECM vs Coll I hydrogels (3 and 7 days), (^#####^ *p* < 0.0001), 2% dKECM vs Coll I (14 days) hydrogels (^###^ *p* < 0.001), and 2% dKECM vs Coll I hydrogels (21 days) (^###^ *p* < 0.001). (B) Water uptake capacity of 2% dKECM and Coll I hydrogels: 2% dKECM and Coll I hydrogels at 0 days vs 21 days (****p < 0.0001).

### 4.3 Assessment of the Bioactivity of the Hydrogels

To evaluate the effectiveness of the dKECM hydrogels as stem cell delivery vehicles, a comprehensive evaluation of cellular bioactivity within the 3D matrix is essential. Assessing metabolic, morphological, and phenotypic parameters provides insights into hydrogel biocompatibility and its influence on the encapsulated cell fate.

#### 4.3.1 Cell Metabolic Activity, Proliferation, and Viability

To evaluate the bioactivity of dKECM hydrogels compared with that of a commercially available option, Coll I hydrogels, we assessed the metabolic activity, proliferation, and viability for 21 days of the hASCs and HK-2 cells (Figure 8). The hASCs encapsulated in dKECM hydrogels exhibited a continuous and statistically significant increase in metabolic activity over 21 days, with notable differences arising after 7 days (Figure 8A,B). In contrast, hASCs in Coll I hydrogels experienced a decrease in metabolic activity after an initial increase, with cell proliferation also decreasing statistically significantly by day 21. Confocal micrographs showed a gradual increase in the number of live hASCs within dKECM and Coll I hydrogels over time (panels C and F, respectively, of Figure 8). A spread-out and elongated morphology for encapsulated hASCs within both hydrogels was observed, suggesting their good adhesion and interaction with the hydrogel’s matrix. A residual cell death was observed initially for both hydrogels, consistent with previous studies.^32,65^ The dKECM hydrogel’s favorable performance is supported by prior research^21^ emphasizing ECM’s role in bioactivity and secretion stimulation.^66^ Both substrates demonstrated a visible degree of contraction, more pronounced for Coll I hydrogels.

**Figure 8:**
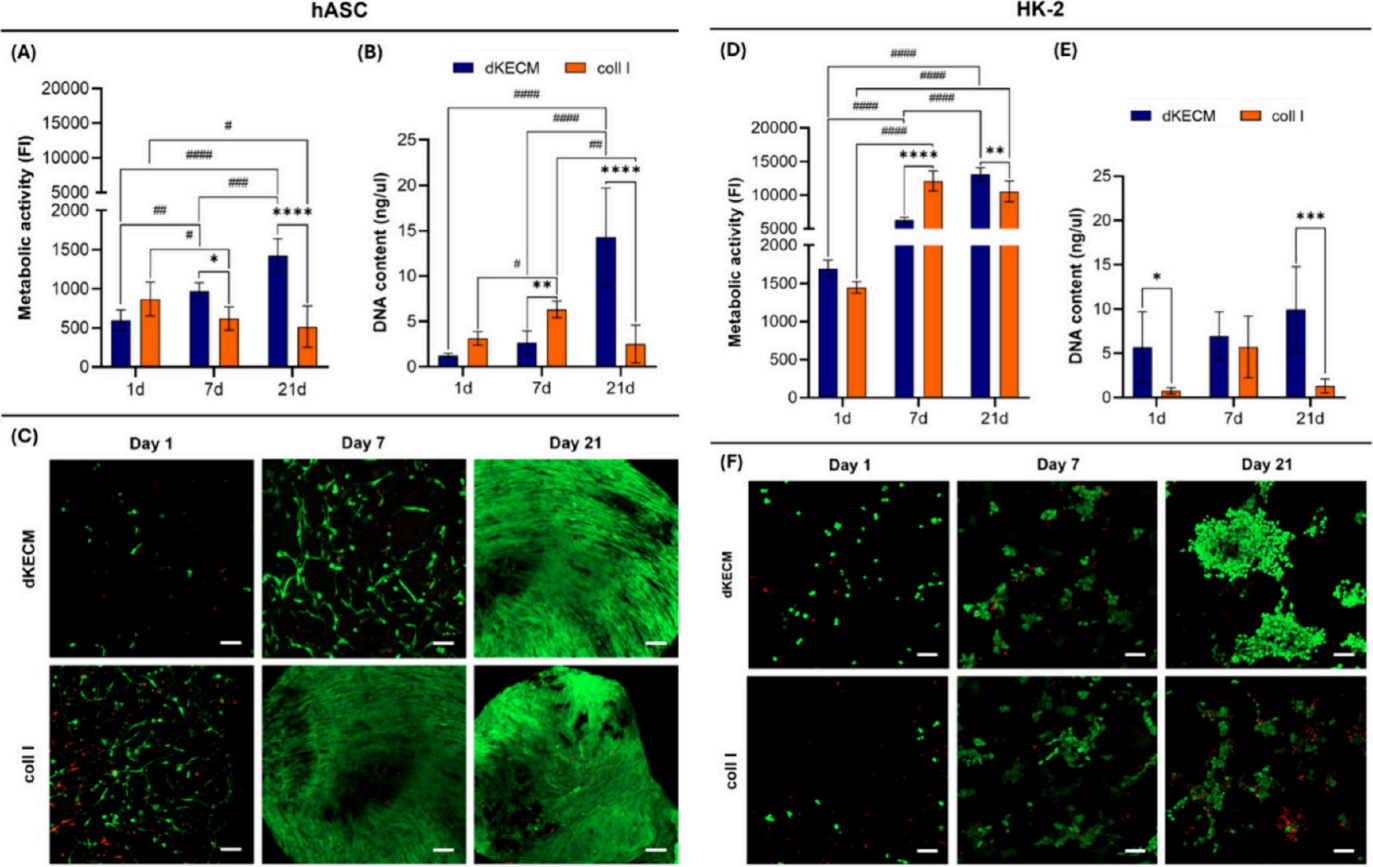
Cytocompatibility assessment of 2% dKECM and Coll I hydrogels in the presence of hASC and HK-2, at 1, 7, and 21 days. (A) Metabolic activity of hASCs. Statistical difference between 2% dKECM hydrogel (1 day vs 7 days, ^##^ *p* < 0.01; 7 days vs 21 days, ^###^ *p* < 0.001; 1 day vs 21 days, ^####^ *p* < 0.0001) and 2% Coll I hydrogel (1 day vs 21 days, ^#^ *p* < 0.05; 1 day vs 7 days, ^#^ *p* < 0.05), and both hydrogels (7 days for dKECM vs 7 days for Coll I, *p < 0.05; 21 days for dKECM vs 21 days for Coll I, ****p < 0.0001). (B) Proliferation of hASC. Statistical difference between 2% dKECM hydrogel (7 days vs 21 days, ^####^ *p* < 0.0001; 1 day vs 21 days, ^####^ *p* < 0.0001) and 2% Coll I hydrogel (1 day vs 7 days, ^#^ *p* < 0.05; 7 days vs 21 days, ^##^ *p* < 0.01), and both hydrogels (7 days for dKECM vs 7 days for Coll I, **p < 0.01; 21 days for dKECM vs 21 days for Coll I, ****p < 0.0001). (C) Imaging of hASCs viability (green for live cells and red for dead cells). (D) Metabolic activity of HK-2 cells. Statistical difference among 2% dKECM hydrogel (1 day vs 21 days, ^####^ *p* < 0.0001; 1 day vs 7 days, ^####^ *p* < 0.0001; 7 days vs 21 days, ^####^ *p* < 0.0001), 2% Coll I hydrogel (1 day vs 7 days, ^####^ *p* < 0.0001; 1 day vs 21 days, ^####^ *p* < 0.0001), and both hydrogels (7 days for dKECM vs 7 days for Coll I, ****p < 0.0001; 21 days for dKECM vs 21 days for Coll I, **p < 0.01). (E) Proliferation of HK-2 cell. Statistical difference between both hydrogels (1 day for dKECM vs 1 day for Coll I, *p < 0.05; 21 days for dKECM vs 21 days for Coll I, ***p < 0.001). (F) Imaging of HK-2 cell viability. Magnification of 10× and scale bar of 100 μm.

HK-2 cells encapsulated in the hydrogels exhibited a metabolic activity that was higher than that of hASCs. As opposed to Coll I hydrogels, dKECM hydrogels supported a consistent increase in the cell’s metabolic activity throughout the culture period of 21 days, with statistically significant increases at all time points (Figure 8D), also corroborated by DNA quantification data (Figure 8E). Coll I hydrogels showed a slight decrease in metabolic activity after 7 days and a decrease in the extent of cell proliferation over time. Although Coll I hydrogels displayed higher cell metabolic activity in the first 7 days, dKECM hydrogels showed significantly enhanced metabolic activity and higher proliferation rates throughout the culture period. Live/dead assays confirmed these trends, with dKECM hydrogels forming large and interconnected aggregates of viable cells by day 21, while Coll I hydrogels showed a high ratio of dead cells around the clusters. Overall, the dKECM hydrogels provide a superior microenvironment for HK-2 cell survival, growth, and proliferation compared to that of Coll I hydrogels, aligning with prior work showing that rich ECM environments stimulate bioactivity.^26^

#### 4.3.2 Cell Morphology

Morphological evaluation of encapsulated cells using H&E and MT staining revealed distinct behaviors on 2% dKECM and Coll I hydrogels (Figure 9). On day 1, hASCs had similar shapes and dispersion in both hydrogels, but their matrix morphology differed due to their composition.^48^ dKECM hydrogels showed extensive, densely packed ECM fibers (Figure 9A), whereas Coll I hydrogels had a smooth, regular structure (Figure 9B). Over time, both hydrogels supported cell proliferation and matrix production. By the end of the culture period, dKECM hydrogels had higher cellular content, and displayed a network of pores and densely packed ECM, facilitating cell spreading. The improved performance of dKECM hydrogels was further corroborated by histological analysis of both hydrogels with HK-2 cells (Figure 9C,D). dKECM hydrogels encapsulating HK-2 cells showed significant structural features, including a progressive increase in the number of cellular clusters forming niches within the matrix. By the end of the culture period, dKECM hydrogels revealed larger and more distributed cell aggregates. These packed cells exhibited a cytoplasmic basolateral surface and organized themselves into cylindrical structures with a central lumen, mimicking the morphology of vascular- and tubule-like structures found in the kidney.^22^ In contrast, HK-2 cells in Coll I hydrogels exhibited minimal cluster formation and underdeveloped structures (Figure 9D). These findings suggest that dKECM hydrogels provide a more favorable environment for HK-2 cell growth and organization compared to Coll I hydrogels.

**Figure 9:**
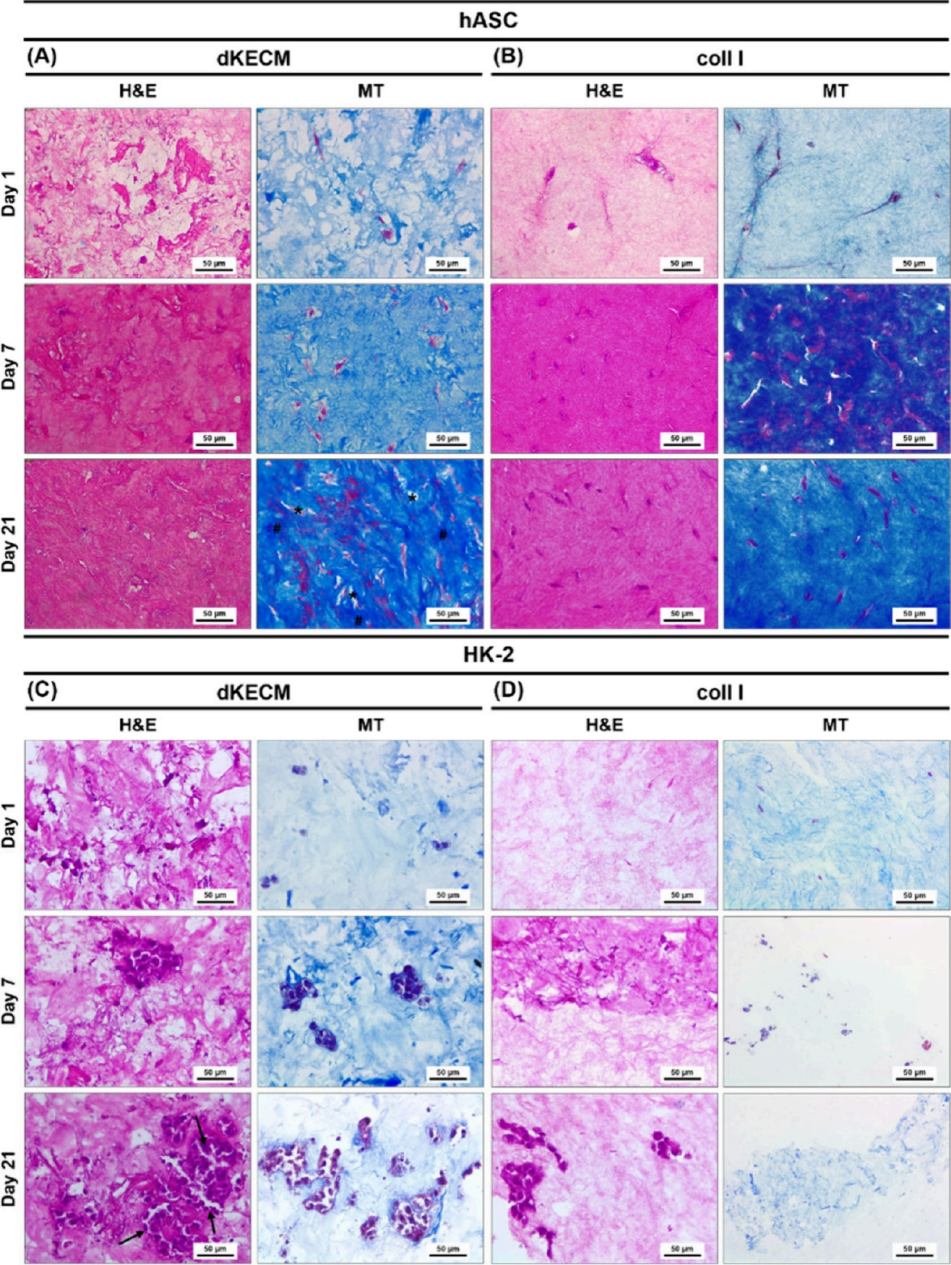
Morphologic evaluation by H&E and MT staining of 2% dKECM and Coll I hydrogels assessed at days 1, 7, and 21 of hASCs (A and B, respectively) and HK-2 cells (C and D, respectively) culture. Resolution of 40× and scale bar of 50 μm.

Histological analysis of both hydrogels revealed that the dKECM hydrogels provided a superior microenvironment for hASC and HK-2 cell growth, activity, and distribution. Unlike Coll I hydrogels, which lack essential proteins like fibronectin,^67^ dKECM hydrogels offer a richer protein composition, promoting enhanced cell spreading and the formation of more mature structures. These findings highlight the potential of dKECM hydrogels as cell carriers for kidney regeneration.

#### 4.3.3 Evaluation of the Cell Phenotype

Immunocytochemistry analyses targeting renal markers were further employed to evaluate the potential of these cell carriers’ systems to influence stem cell differentiation, throughout 21 days (Figure 10). CD133, WT1, and PAX2 markers were used to evaluate immature cells,^41^ early to intermediate differentiation stages,^68^ and renal epithelial lineage commitment, respectively^32^ (Figure 10A–C). No CD133, a membrane glycoprotein primarily located in the cell membrane, was detected in hASCs encapsulated in either hydrogel (Figure 10A). WT1, indicative of kidney progenitor cells and epithelial precursors, was also absent from both hydrogels (Figure 10B). However, strong PAX2 nuclear staining was observed in hASCs within dKECM hydrogels after 21 days, suggesting enhanced commitment to the renal epithelial lineage compared to Coll I hydrogels (Figure 10C).^32^ To assess kidney lineage differentiation, nephrin and podocin were selected as podocyte development markers (panels D and E, respectively, of Figure 10), while sodium-glucose cotransporter 2 (SGLT2) and aquaporin-1 (AQP1) were used to evaluate tubular differentiation (panels F and G, respectively, of Figure 10). Indeed, SGLT2 and AQP-1, membrane proteins in renal tubular cells, work together to regulate water and solute balance.^69^ dKECM hydrogels displayed stronger localized signals for these markers, with peak intensity at day 7 (panels D and E, respectively, of Figure 10). Coll I hydrogels followed a similar pattern for nephrin but showed a continuous increase in the level of podocin staining throughout the culture period. dKECM hydrogels with hASCs showed well-defined AQP-1 staining after 7 days, persisting throughout the experiment (Figure 10F). In contrast, AQP-1 in Coll I hydrogels was present only on the first day and became undetectable thereafter. Similarly, SGLT2 expression in dKECM hydrogels was present on both days 1 and 21 (Figure 10G), whereas Coll I hydrogels showed only residual staining throughout the culture period. These results demonstrate that hASCs in dKECM hydrogels begin expressing renal markers early, with sustained differentiation toward tubular and podocyte phenotypes after a week. The stronger and persistent expression of AQP-1 and SGLT2 in dKECM hydrogels, compared to that in Coll I hydrogels, suggests a greater potential for tubular lineage differentiation.

**Figure 10:**
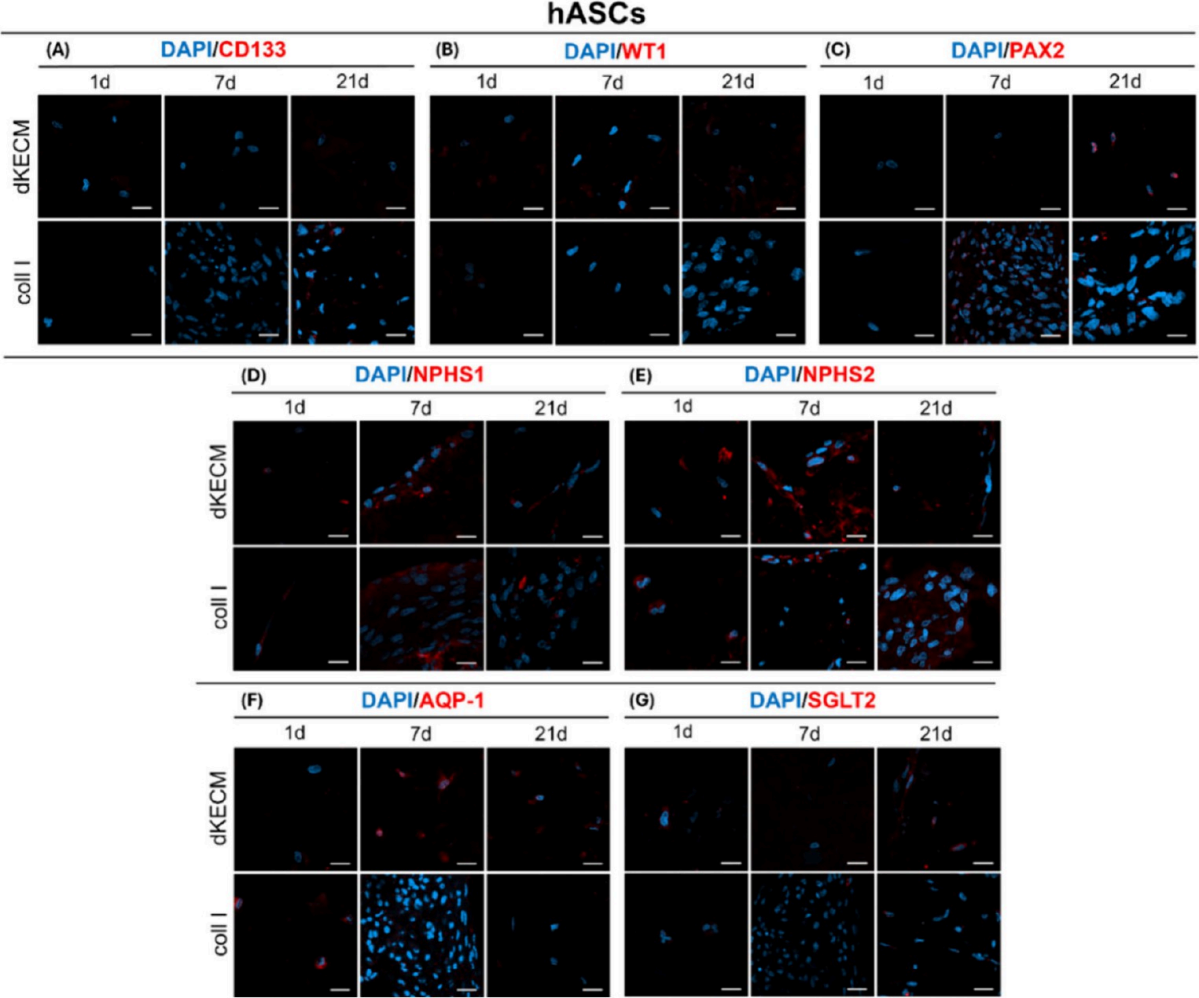
Phenotypic evaluation of hASCs encapsulated into 2% dKECM and Coll I hydrogels by immunofluorescence. DAPI staining (blue) was used to visualize the nuclei of cells. hASCs were stained for specific renal markers (red), which include markers for kidney development: (A) CD133, (B) Wilms’ tumor 1 (WT1), (C) paired box gene 2 (PAX2). Podocyte differentiation markers: (D) nephrin (NPHS1) and (E) podocin (NPHS2). Proximal tubular markers: (F) aquaporin-1 (AQP-1) and (G) sodium-glucose cotransporter 2 (SGLT2). Magnification of 63× and scale bar of 20 μm.

The contrasting effects of the hydrogels were more pronounced on adult renal cell behavior than on stem cells. As expected, due to their mature state, HK-2 cells did not show significant expression of stem cell markers CD133 and WT1 (panels A and B, respectively, of Figure 11) in either dKECM or Coll I hydrogels. However, strong nuclear PAX2 expression was observed in dKECM hydrogels from day 1, persisting throughout the experiment (Figure 11C). While PAX2 expression is already known to occur during tubular differentiation,^32^ its presence from day 1 suggests that dKECM hydrogels may create a unique microenvironment, promoting initial nephron differentiation steps in encapsulated HK-2 cells. dKECM hydrogels consistently displayed markers for both podocytes (NPHS1 and NPHS2) and tubular epithelial cells (AQP-1 and SGLT2) over the 21 day culture period (Figure 11D–G). AQP-1 expression was strongest after 7 days, while other markers remained visible throughout. In contrast, HK-2 cells in Coll I hydrogels showed the transient presence of AQP-1 on day 7, with other markers not sustained. Although HK-2 cells are mature proximal tubule cells,^70^ the detection of podocyte markers in dKECM hydrogels suggests an influence on cellular behavior. dKECM hydrogels promoted early nephron differentiation and sustained marker expression in HK-2 cells, whereas Coll I hydrogels had minimal differentiation and significant cell loss.

**Figure 11:**
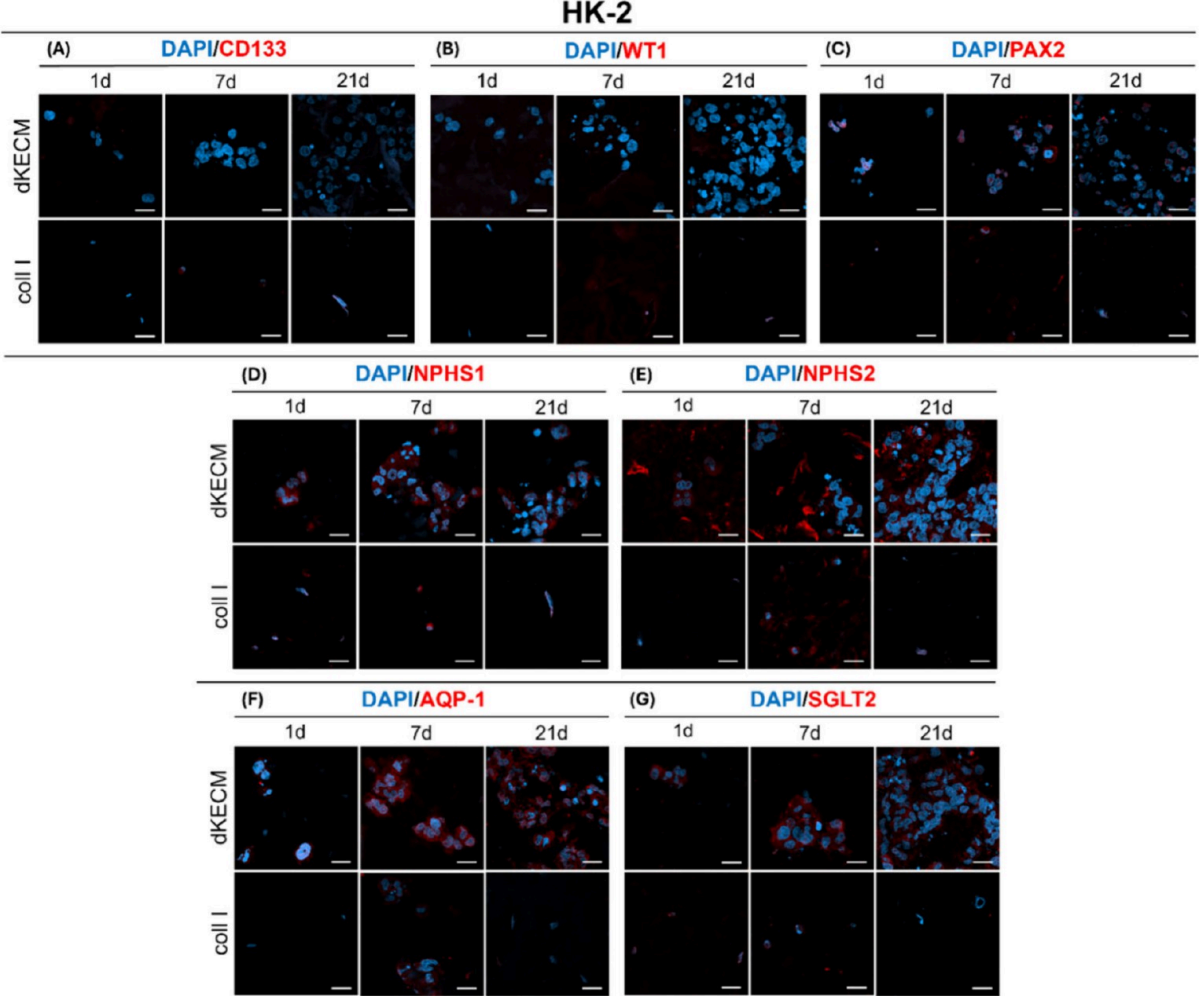
Immunofluorescence micrographs of HK-2 cells encapsulated into 2% dKECM and Coll I hydrogels on days 1, 7, and 21. DAPI (blue) was used to stain cell nuclei, while fluorescent antibodies (red) were employed to evaluate the expression of target renal markers, including (A) CD133, (B) Wilms’ tumor 1 (WT1), (C) paired box gene 2 (PAX2), (D) nephrin (NPHS1), (E) podocin (NPHS2), (F) aquaporin-1 (AQP-1), and (G) sodium-glucose cotransporter 2 (SGLT2). Magnification of 63× and scale bar of 20 μm.

These results indicate that the rich ECM composition of dKECM hydrogels not only supports cell growth and proliferation but also provides effective biological cues, which trigger signaling cascades that influence gene expression and ultimately drive cell differentiation.

## 5 Conclusion

Our study successfully highlights the suitability of dKECM hydrogels as bioactive scaffolds for stem cell-based therapy for CKD. Through effective decellularization, dKECM hydrogels were shown to preserve important aspects of the native ECM, influencing cellular behavior and phenotype maintenance. Our results demonstrate that dKECM hydrogels outperform Coll I hydrogels by providing a microenvironment more akin to native ECM, rich in essential biochemical and structural components. This enriched microenvironment better supports cell viability, proliferation, and metabolic activity over time. Moreover, HK-2 cells encapsulated into dKECM hydrogels exhibited organized cluster formation and maintained renal cell characteristics more effectively compared with those in Coll I hydrogels.

Overall, the dKECM hydrogels effectively replicated the kidney-specific microenvironment, promoting the spreading and differentiation of stem cells into renal lineages while also maintaining the renal phenotype in adult renal cells. These findings underscore the importance of considering ECM structure and bioactivity in the design of injectable biomaterials as stem cell carrier systems, particularly for applications in renal tissue regeneration. Future research should further elucidate the molecular mechanisms underlying the observed cellular responses and explore the *in vivo* potential of dKECM hydrogels for therapeutic interventions aiming to restore renal function.

## Supplementary Material



## Notes

Supplementary rheological characterization, including shear rate ramp analysis, of 2% dKECM and Coll I hydrogels (PDF)
