# Supplementary material for: Kidney-Derived ECM Hydrogels as Cell Delivery Devices
Source: ACS Appl Mater Interfaces. 2025 Mar 10;17(11):16421–36. doi: 10.1021/acsami.4c15873 (PMC12772458; doi:10.1021/acsami.4c15873)
Supplement: Supplementary file 1 [file am4c15873_si_001.pdf]

Supporting Information

Kidney-derived ECM hydrogels as cell delivery devices

Ana M. Rodrigues [a,b], Sara Gimondi[a,b], Rita Quinteira[a,b], Helena Ferreira[a,b], Albino Martins[a,b], Nuno M. Neves[a,b]*

[a] 3B's Research Group, I3Bs - Research Institute on Biomaterials, Biodegradables and Biomimetics of University of Minho, AvePark -, Parque de Ciência e Tecnologia, Rua Ave 1, Edificio 1 (Sede), Barco, Guimarães, 4805-694, Portugal;

[b] ICVS/3B's - PT Government Associate Laboratory, Braga/Guimarães, Portugal

*Email: nuno@i3bs.uminho.pt

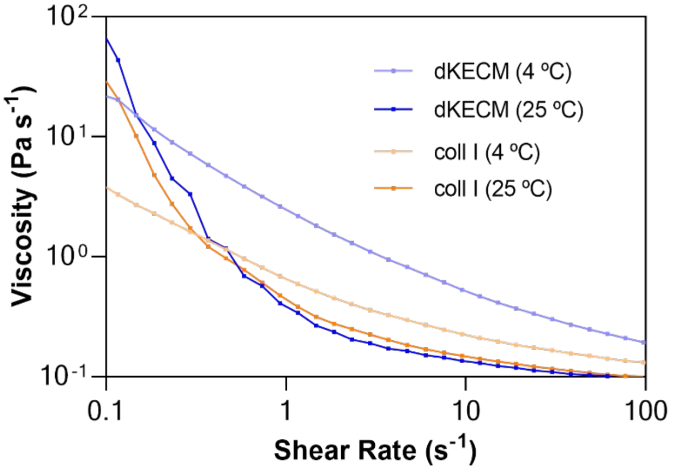

Figure S. 1. Rheological characterization of 2% dKECM and coll I hydrogels. Shear rate ramp from 0.1 to 100 s^{-1}, for 2 minutes to determine the viscosity of both hydrogels at 4 and 25 ºC.
